# Successful recording of direct cortical motor-evoked potential from a pediatric patient under remimazolam anesthesia: a case report

**DOI:** 10.1186/s40981-022-00555-y

**Published:** 2022-08-22

**Authors:** Kotoe Kamata, Suguru Asagi, Yoshiteru Shimoda, Masayuki Kanamori, Nozomu Abe, Shigekazu Sugino, Teiji Tominaga, Masanori Yamauchi

**Affiliations:** 1 Department of Anesthesiology and Perioperative Medicine, Tohoku University School of Medicine, 2-1 Seiryo-machi, Aoba-ku, Sendai-shi, Miyagi 980-8575 Japan; 2 Clinical Physiological Center, Tohoku University Hospital, 2-1 Seiryo-machi, Aoba-ku, Sendai-shi, Miyagi 980-8575 Japan; 3 Department of Neurosurgery, Tohoku University School of Medicine, 2-1 Seiryo-machi, Aoba-ku, Sendai-shi, Miyagi 980-8575 Japan

**Keywords:** Direct cortical motor-evoked potential, Pediatric, Anesthesia, Remimazolam

## Abstract

**Background:**

Intraoperative motor-evoked potential (MEP) monitoring reduces postoperative motor deficits. Propofol-based total intravenous anesthesia is the gold standard for intraoperative myogenic MEPs. Although there is no contraindication to administering propofol in adults with peanut, soy, or egg allergies, its safety in children with these allergies remains unclear.

**Case presentation:**

A 12-year-old girl required general anesthesia under intraoperative direct cortical MEP (dc-MEP) monitoring due to supratentorial glioma. Remimazolam-based anesthesia was selected, instead of propofol, due to the patient’s egg hypersensitivity. Stable myogenic MEPs were recorded throughout the surgery with remimazolam at 0.9 mg/kg/h and remifentanil at 0.35 μg/kg/min, following adjustments of stimulation intensity and titration of remimazolam infusion. Neither intraoperative memory nor motor deficits were present after surgery.

**Conclusions:**

We present a pediatric case whose dc-MEP was recorded under remimazolam anesthesia. The cardiovascular stability and avoidance of propofol infusion syndrome with remimazolam were superior to propofol.

## Background

The pediatric population has more risk of neurological deterioration during neurosurgical procedures than adults; thus, intraoperative neurophysiological monitoring is beneficial [1]. Intravenous anesthetics, especially propofol, are preferable when myogenic motor-evoked potential (MEP) monitoring is required due to its much less interference with alpha motor neuron excitability than inhalational agents [2]. Although no connection between propofol allergy and egg, soy, or peanut allergies were found in adults [3], it is still unclear whether propofol administration is safe in children allergic to certain foodstuffs [4]. Remimazolam, a short-acting benzodiazepine characterized by metabolism independent of organ function [5], was proposed as an alternative to propofol for spine surgeries in young and elderly patients who require intraoperative transcranial MEP (tc-MEP) recordings [6, 7]. This study presents a pediatric case with egg hypersensitivity whose direct cortical MEP (dc-MEP) was successfully recorded under remimazolam anesthesia, instead of propofol. This case report was prepared following the ACRE checklist developed from the ACRE guideline [8].

## Case presentation

A 12-year-old girl (weight 55 kg; height 1.5 m; ASA-PS Class 2) was referred to our institution with a right parietotemporal tumor. The tumor was diagnosed as anaplastic astrocytoma via stereotactic biopsy and was refractory to chemoradiotherapy; thus, partial resection was planned. Intraoperative dc-MEP was essential since the medullary arteries located deep in the sulcus supply the pyramidal tract. Preoperative motor function of the patient's extremities was normal, with a manual muscle testing score of 5. Because the preoperative allergy test showed egg hypersensitivity, remimazolam-based general anesthesia, instead of propofol, was selected. Anti-epileptic therapy with levetiracetam (2000 mg daily) was the sole medication before surgery.

Besides standard ASA monitoring, invasive arterial pressure and processed electroencephalogram (EEG) monitoring were adopted. General anesthesia was induced with remimazolam at 6 mg/kg/h and remifentanil at 0.5 μg/kg/min. After the loss of consciousness, remimazolam infusion was reduced to 1.5 mg/kg/h, then 30 mg of rocuronium was given to facilitate endotracheal intubation. Neither additional rocuronium nor sugammadex was administered during MEP monitoring. Remifentanil dose was adjusted between 0.3 and 0.4 μg/kg/min according to surgical stimuli. The infusion rate of remimazolam was titrated to attain a Bispectral Index® (BIS; Medtronic-Covidien, Dublin, Ireland) value of 60 using an electrode placed upon the forehead of the contralateral side of the tumor.

**Fig. 1:**
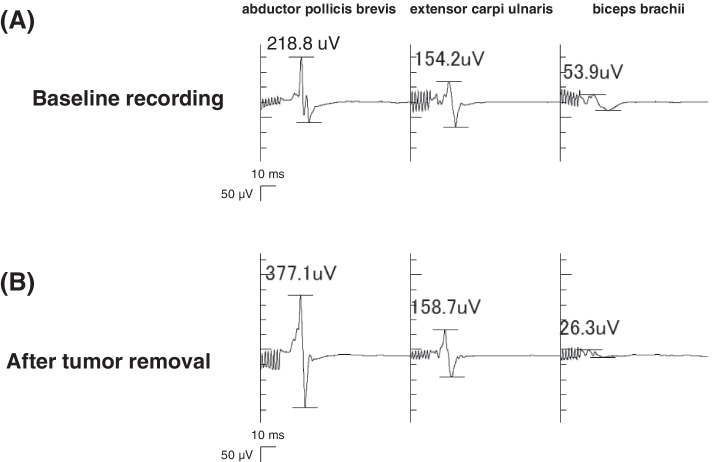
MEPs from the left upper extremities upon direct cortical stimulation at the baseline recording (**A**) and after the tumor removal (**B**). The numbers superimposed show MEP amplitude. Stable myogenic MEPs were recorded throughout the surgery (**A**, **B**) with intravenous infusions of remimazolam at 0.9 mg/kg/h and remifentanil at 0.35 μg/kg/min

After craniotomy and durotomy, a strip electrode was positioned subdurally onto the cortex. Monitoring of dc-MEP was performed using one of the contacts of the strip electrode as the anode, whereas an electrode positioned above the nasion at Fpz according to the 10–20 International System was used as the cathode. To detect the compound muscle action potentials, seal-type electrodes were placed on the relevant muscles of the contralateral side of the tumor in a standardized way: the upper extremities (abductor pollicis brevis, extensor carpi ulnaris, biceps brachii, and deltoid) and the lower extremities (abductor hallucis, tibialis anterior, and quadriceps). Continuous MEP monitoring by monophasic direct cortical stimulation was performed using a neurophysiological monitoring device (Neuromaster MEE-1216, Nihon Kohden, Tokyo, Japan) with a 5 Hz –1.5 kHz bandpass. Direct cortical stimulation by train-of-five pulses with interstimulus intervals of 2 ms at 15 mA with a pulse duration of 0.5 ms was delivered by subdural electrodes. Because no significant motor responses were observed, electrode locations and stimulation intensity were adjusted. In addition, the initial rate of remimazolam administration of 1.8 mg/kg/h varied incrementally to 0.8 mg/kg/h, with careful observation of BIS values as well as raw EEG waveforms, while remifentanil infusion remained at 0.3 μg/kg/min. Then, the patient’s motor responses gradually appeared with interstimulus intervals of 2 ms at 20 mA. Finally, body movement was elicited by train-of-five pulses with interstimulus intervals of 2 ms at 25 mA. To prevent false-negative signals, MEPs were recorded throughout the tumor removal as the constant-current stimulation with the suprathreshold intensity by train-of-seven pulses with interstimulus intervals of 2 ms at 23 mA from the upper extremities. The bridging veins hindered electrode placement for lower extremities monitoring. A successful dc-MEP recording was defined as detecting a certain compound muscle potential apparent from the background activity. Combination of remimazolam at 0.9 mg/kg/h and remifentanil at 0.35 μg/kg/min provided stable MEP recordings (Fig. 1A). The blood pressure remained within 20% of the baseline level without any drug administration. Body temperature, ventilation status, and hematocrit level were kept within the physiological ranges. After the surgery, the patient regained consciousness and was extubated 8 min after remimazolam termination. A total of 829 mg of remimazolam and 14.5 mg of remifentanil were administered. The total duration of the surgery and anesthesia was 601 min and 735 min, respectively. No significant dc-MEP changes were observed relative to baseline values during the tumor removal for 215 min (Fig. 1B). The patient showed moderate sensory disturbance in the left upper limb due to postcentral gyrus removal, but neither motor deficits nor memory of the operation was present.

## Discussion

This is the first case report in which dc-MEP was successfully recorded in a 12-year-old neurologically immature patient using remimazolam. Intraoperative neurophysiological monitoring used for supratentorial surgery has been investigated mainly for adults, despite recording myogenic MEP as a valuable safety measure in complex intracranial surgery when the lesion is in proximity to the motor cortex and fibers [9]. However, the parameters for cortical stimulation need to be adjusted in young children and infants due to their central nervous system immaturity [10]. Age significantly impacts cortical development; Eyre et al. indicated that a higher stimulation threshold to elicit a motor response is necessary for children up to 16 years old [11]. Furthermore, the decreased interhemispheric transmission time of evoked potential along with increasing age from 7 to 17 years reported by Hagelthorn et al. suggests that increasing corpus callosal myelination during late childhood integrates functional connection across the midline [12]. Spinal cord motor pathways also require a prolonged period of maturation. Unlike other pathways, the corticospinal tracts (CSTs) undergo a protracted period of myelogenesis and synaptogenesis; the diameters of corticospinal axons do not reach full myelination until the age of 16 [13]. Nezu et al. estimated that electrophysiologic maturity of the CSTs innervating the hand muscles is completed by the age of 13 [14]. Moreover, direct cortical stimulation is sometimes required in supratentorial surgeries because the stimulating electrodes in tc-MEP, placed overlapping the motor areas on the scalp, could interfere with the surgical field. Therefore, much lower current intensities than transcranial electrical stimulation are adopted for direct motor cortex stimulation. Notably, the titration of remimazolam infusion and the adjustment of monitoring conditions in our case were complicated. However, a recent report on pediatric supratentorial surgeries showed that dc-MEP was more sensitive than tc-MEP in predicting postoperative motor decline despite the patients being aged 3–207 months [15]. The fact that persistent loss of MEP response for more than 5 min results in postoperative motor deficit [2] also encourages us to find more reliable and effective conditions for motor potential monitoring, even if the patient is not neurologically matured.

MEP is sensitive to neurological conditions, the extent of myelination of corticospinal pathways, and anesthesia techniques. Clinically recommended doses of opioids, such as fentanyl or remifentanil, have little impact on MEPs [16]; however, the anesthesia plan must be carefully considered because the choice of anesthetic affects the likelihood of a false-positive result. In order to record reliable MEPs, total intravenous anesthesia with propofol, rather than inhalational agents, is recommended in children over 6 years old [17]. Propofol has advantages over other intravenous agents, such as ketamine or midazolam; it exhibits higher suitability for EEG changes to the depth of anesthesia, and a well-established target-controlled infusion system can precisely control propofol concentration. Nevertheless, propofol involves a potential risk for propofol infusion syndrome (PRIS), originally found in children but has become often reported in adults even within the recommended limits [18]. The demand for an alternative anesthesia regimen for the ketamine-based technique in children younger than 6 years old [17] and propofol dose-dependent suppression of MEP amplitude [19] are of significant concern. Considering the effects of physiological variables during MEP, a significant decrease in systemic arterial pressure caused by propofol seems inferior to remimazolam. Doi and colleagues revealed that hypotensive events were more frequent with propofol than with remimazolam during all phases of the anesthesia. The requirement for vasopressors was greater for propofol than for remimazolam [20]. Further research is still required to determine propofol safety in pediatric cases with severe anaphylaxis to eggs and allergic reactions to peanut or soy [4]. In this pediatric case, we avoided propofol due to the patient’s history of egg allergy and decided to administrate remimazolam. The clinical use of remimazolam is increasing worldwide. This case report demonstrates that dc-MEP recording under remimazolam anesthesia is possible even if the patient’s neurological development is not maturated.

Remimazolam has some issues that should be addressed. First, the reliability of frontal EEG-derived index alteration is still controversial [21], despite the good correlations between the BIS value, and the effects of propofol, midazolam, and isoflurane on the level of consciousness and recall were evident [22]. The EEG effects of benzodiazepines are predominantly a monotonic beta activation, especially in frontal areas, and these effects map to higher EEG-derived indices to estimate the anesthesia depth [23]. In contrast to the current pediatric case, previous tc-MEP reports of young and elderly patients showed that relatively higher remimazolam infusion rates were required for successful MEP recording [6, 7]. In neurosurgery, stimulating intensities should be minimized to the least possible extent to circumvent false-positive signals that result from the stimuli reaching the skull base [16]; this is the most significant difference from tc-MEP, which is frequently used in spine surgery. Certain indicators to estimate the level of remimazolam anesthesia are required to make the minimum infusion rate compatible with avoiding intraoperative awareness. Long-term benzodiazepine use for seizure control, frequently observed among patients with intracranial legions, is another concern; otherwise, the patient might show remimazolam tolerance [24], and reversal with flumazenil may cause a seizure. Thus, at present, eliminating the incidences of intraoperative hypotension or PRIS is the most rational reason for considering remimazolam-based anesthesia for pediatric supratentorial surgery under MEP monitoring.

The present case report had some limitations. First, it is still unclear whether remimazolam suppresses dc-MEP responses. A previous report could not find any dose-dependent effects of remimazolam on tc-MEP [6], though several reports indicated the progressive suppression of tc-MEP with the increase of midazolam dose [25, 26]. In our case, a successful dc-MEP recording was obtained with a reduced remimazolam infusion. Second, the pharmacological differences between midazolam and remimazolam on MEP are considered clinically important, but this study could not investigate these differences based on the experience of a single case handled with remimazolam anesthesia. Further studies are needed to address these limitations.

## Conclusions

Remimazolam can be used as an alternative to propofol for dc-MEP monitoring even if the patient is presumed neurologically immature. Maintaining arterial blood pressure and avoiding PRIS are advantages of using remimazolam instead of propofol.

## Data Availability

The datasets used and analyzed during the current case are available from the corresponding author upon request.

## Abbreviations

ASA: American Society of Anesthesiologists
BIS: Bispectral index
CST: Corticospinal tract
dc-MEP: Direct cortical MEP
EEG: Electroencephalogram
MEP: Motor-evoked potential
PRIS: Propofol infusion syndrome
tc-MEP: Transcranial MEP
